# On the representation of cells in bone marrow pathology by a scalar field: propagation through serial sections, co-localization and spatial interaction analysis

**DOI:** 10.1186/s13000-015-0383-0

**Published:** 2015-09-02

**Authors:** Cleo-Aron Weis, Benedict Walter Grießmann, Christoph Scharff, Caecilia Detzner, Eva Pfister, Alexander Marx, Frank Gerrit Zoellner

**Affiliations:** Institute of Pathology, University Medical Centre Mannheim, Heidelberg University, Theodor-Kutzer-Ufer 1-3, 68167 Mannheim, Germany; Department of Nephropathology, Institute of Pathology, University Hospital Erlangen, Friedrich-Alexander-University Erlangen-Nuermberg, Erlangen, Germany; Computer Assisted Clinical Medicine, University Medical Centre Mannheim, Medical Faculty Mannheim, Heidelberg University, Mannheim, Germany

## Abstract

**Background:**

Immunohistochemical analysis of cellular interactions in the bone marrow in situ is demanding, due to its heterogeneous cellular composition, the poor delineation and overlap of functional compartments and highly complex immunophenotypes of several cell populations (e.g. regulatory T-cells) that require immunohistochemical marker sets for unambiguous characterization. To overcome these difficulties, we herein present an approach to describe objects (e.g. cells, bone trabeculae) by a scalar field that can be propagated through registered images of serial histological sections.

**Methods:**

The transformation of objects within images (e.g. cells) to a scalar field was performed by convolution of the object’s centroids with differently formed radial basis function (e.g. for direct or indirect spatial interaction). On the basis of such a scalar field, a summation field described distributed objects within an image.

**Results:**

After image registration i) colocalization analysis could be performed on basis scalar field, which is propagated through registered images, and - due to the shape of the field – were barely prone to matching errors and morphological changes by different cutting levels; ii) furthermore, depending on the field shape the colocalization measurements could also quantify spatial interaction (e.g. direct or paracrine cellular contact); ii) the field-overlap, which represents the spatial distance, of different objects (e.g. two cells) could be calculated by the histogram intersection.

**Conclusions:**

The description of objects (e.g. cells, cell clusters, bone trabeculae etc.) as a field offers several possibilities: First, co-localization of different markers (e.g. by immunohistochemical staining) in serial sections can be performed in an automatic, objective and quantifiable way. In contrast to multicolour staining (e.g. 10-colour immunofluorescence) the financial and technical requirements are fairly minor. Second, the approach allows searching for different types of spatial interactions (e.g. direct and indirect cellular interaction) between objects by taking field shape into account (e.g. thin vs. broad). Third, by describing spatially distributed groups of objects as summation field, it gives cluster definition that relies rather on the bare object distance than on the modelled spatial cellular interaction.

**Electronic supplementary material:**

The online version of this article (doi:10.1186/s13000-015-0383-0) contains supplementary material, which is available to authorized users.

## Background

Histological interpretation of lympho-hematopoietic tissues (e.g. bone marrow, lymph nodes, thymus) is a demanding task in many haematological diseases due to a highly complex composition of these tissue comprising lymphoid, myeloid, dendritic and eventually epithelial cells and bony structures in addition to notoriously “fuzzy borders” even of well defined functional structures (e.g. lymphoid follicles, niches [1–6]). Against this background, several intricate issues need to be addressed [7, 8]: a) The quantitative evaluation of distinct cell populations per area, e.g. CD4+, CD25+, Foxp3+ regulatory T-cells (Tregs) [9] that may need a set of immunohistochemical markers for identification. b) The spatial distribution of different cell populations in relation to each other and c) to functional regions (e.g. paratrabecular or perivascular niches) [7, 8].

In the routine diagnostic setting, the issues are currently addressed by rough visual estimation of cellular contents and locations. Reliable counting of cellular infiltrates and manual delineation of regions in combination with sophisticated multiplex immunohistochemical staining or confocal microscopy are usually reserved to scientific questions.

By exploring the bone marrow histology in chronic myeloid leukaemia (CML) with regard to the immunological milieu in the context of an on-going study [10], we have been facing all of the above-mentioned issues. Niches that are supposed to harbour leukaemia stem cells [5] and the tumour microenvironment that comprises mesenchymal stromal cells and various immune cells are of particular interest [11] in relation to the hypothetical impact of immunity on the eradication of CML and the modulation of the immunological milieu by antibodies [12] and other drugs (e.g. kinase inhibitors) [13, 14].

To more objectively describe the complex cellular composition of the bone marrow in CML and the interaction of cell populations that need definition by a plethora of immunohistochemical markers, we herein propose a method to annotate cells or rather cell cluster by scalar fields and to propagate these fields through several registered images. By doing so, quantification (a), localization in regard to niches (b) and spatial interaction analysis (c) could be addressed achieved.

## Methods

### Patient collective

For this technically oriented, proof-of-principle study we used two instructive cases of formalin-fixed bone marrow (trephine) biopsies from the archive of the Institute of Pathology, University Medical Centre Mannheim: One depicts the classical paratrabecular infiltrates of a follicular lymphoma; the other one shows scattered lymphoid cells among the dense granulocytic infiltrates in a case of CML.

### Immunohistochemistry staining

For immunohistochemistry (IHC) staining, the following antibodies were applied on formalin-fixed, paraffin-embedded tissue using a routine immunoperoxidase technique [15, 16] and experimental sequential IHC [17]: anti-CD3 (Dako M7254), anti-CD4 (Dako M7319), anti-CD8 (Dako M7103), anti-CD20 (Dako M0755), anti-Bcl2 (DakoM0887), anti-MPO (Dako C7246), anti-CD61 (Dako C7280). Two chromogens were used, DAB (DAB chromogen) as fix and NovaRed (VECTOR NovaRed) as removable chromogen for sequential IHC [17].

### Comment on image ethics

The histological images shown and used within this work underwent image registration and cropping as part of the method described herein. However, image compression was not applied and image augmenting or manipulating techniques were not performed (e.g. contrast enhancement, gamma setting) according to Digital Image Ethics [18–20].

### Image acquisition and pre-processing (Additional file 1: Figure S1)

Differently stained histological slides of bone marrow biopsies were fully digitalized using an Aperio ScanScope (Aperio/Leica biosystems) and saved in the proprietary svs-format. These files (circa 1GB per file) were decomposed into two regions (each composed of 11,000x10,000 pixel, i.e. about 450 MB per file) and saved in the tagged image file format (tiff) to be manageable with a standard desktop computer. A pathologist approximately chose the regions of interest in serial sections (= rough manual matching).

Then, all files per case were loaded into Fiji [21] and registered with several, established plugin-functions (“Register Virtual Stack Slices” [22, 23], „Template Matching” [24], „StackReg” [25, 26]). Also in Fiji, a colour deconvolution plugin (“Colour Deconvolution” [27–29]) was used to separate the brown (immunoperoxidase staining) and blue areas (DAB background staining) per image. Subsequently, these registered image sets were transferred to MATLAB (version R2014a, Mathworks, Natick, MA, USA).

### Definition and segmentation of spaces and niches

In bone marrow histology, there are several spaces that are defined via their distance to certain easily recognizable structures e.g. paratrabecular or perivascular spaces. The cellular composition of these spaces and the infiltration by certain neoplastic populations in the course of haematological diseases (e.g. follicular lymphoma, CML) are of high diagnostic relevance [7]. Some of these anatomic regions overlap with functional microenvironments for stem cells called niches [3, 5, 30].

In the current study, we focused on the paratrabecular region for the sake of simplicity. This was achieved by manually segmenting in Fiji the bone trabeculae (in the images within this work visualized as green area) and their subsequent description as scalar field by the convolution approach described below.

### Image processing in MATLAB to detect cells (Additional file 1: Figure S1)

Registered images were loaded into the MATLAB environment and underwent further processing by custom-arranged standard image processing procedures. (link to a corresponding Git-repository: https://bitbucket.org/CATWeis/on-the-representation-of-cells-in-bone-marrow-pathology-by-a): image segmentation (application of thresholding algorithms), object detection (application of cross-correlation algorithms) and separation (application of watershed algorithms). At the end a detected nucleus/particle is represented by a centroid. Centroids that are within a certain range (application of distance measure algorithm) to immunoperoxidase-stained areas are referred as “stained” whereas the remaining are referred as “non-stained”.

### Introduction of a field (Additional file 1: Figure S1)

The basic assumption of this work is that every object (cells, bone trabeculae etc.) can be represented by a scalar field with its maximum at the object’s centroid. Mathematically, this approach is related to multidimensional kernel estimation [31, 32].

**Fig. 1:**
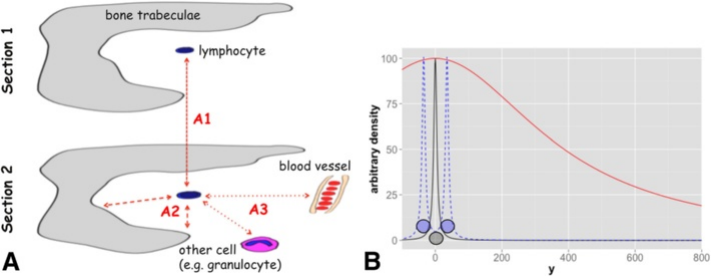
Illustration of the task and the solution via a RBF. **a**: Sketch of two serial sections where a single lymphocyte in the centre could be appreciated in both sections. (A1) One task is to colocalize this cell/its immunohistochemical staining in both sections. (A2) Another task is to measure its distance to the bone trabeculae. (A3) Furthermore, is should be allocated to niches via measuring its distance to certain objects (bone trabeculae, other cells, vessels). **b**: By usage of different parameters (β and γ in equation 1) a RBF for direct and indirect spatial interaction could be modelled according to the particular image resolution/pixel-distance relation. The parameters are here chosen to accomplish a shape for RBF_direct_, which represents potential direct object-object interaction, and for RBF_indirect_, which represents paracrine interaction. Black solid line: One point at position x_p_ = 0 (representing a nucleus) is convoluted with an inverse, multiquadric basis function for direct interaction (RBF_direct_ with β = 3 and γ = 0.01). For illustration purposes the RBF is only shown with one variable. As a consequence, every point in this figure (xЄ [−100,800]) has an arbitrary scalar field value [arbitrary density]. Blue dashed line: Overlay of two fields for direct interaction, where one represents a centroid at x_p_ = −50 and one at x_p_ = 50.Red solid line: The single point at position x_p_ = 0 is convoluted with an inverse, multiquadric basis function for indirect interaction (RBF_indirect_ with β = 3 and γ = 0.00002)

The scalar field of an object is represented by a radial basis function (RBF), in this work, by an inverse multiquadric RBF

1 $$ \mathrm{C}\left(\mathrm{x}, \mathrm{y}\right)=\upalpha \sqrt{\frac{1}{\upbeta +\upgamma *{\left(\mathrm{x}-{\mathrm{x}}_{\mathrm{P}}\right)}^2}+\frac{1}{\upbeta +\upgamma *{\left(\mathrm{y}-{\mathrm{y}}_{\mathrm{P}}\right)}^2}} $$

with x and y as variables and with x_P_ and y_P_ as coordinates of the centroid. The invers multiquadric RBF was chosen among the many other RBF due to its feasible shape for this project and due to its many changeable parameters. However, many other RBF would be likewise applicable. The parameter α is chosen to have an arbitrary maximum of 100 [arbitrary unit] at the coordinates of the centroid. Hereby, the shape of the RBF, which is defined by the parameters $\upbeta$ and $\upgamma$, could be adapted to different cell interaction models [33]: Hypothesis 1) Direct spatial cell-cell- and cell-niche-interaction (henceforth called ‘direct interaction’) is represented by a RBF (henceforth denoted by ’RBF_direct_’) with high values in the area of the nucleus (>90), mediate values in the area of the cell (>80) and with a step, asymptotic decrease towards 0 (black solid line Fig. 1). Hypothesis 2) Indirect spatial interactions via proposed secretory factors (henceforth called ‘indirect interaction’) are represented by a RBF (henceforth denoted by ‘RBF_indirect_’) with, again, high values at the centroid position (>90) but a broad shape and medium values within the proposed 250 μm range for paracrine interaction [33, 34] (solid red line Fig. 1).

On the basis of this function, for every image a kernel (application of frequency space convolution algorithm) is calculated that needs to have the size of the image and that must not have the value 0 at any point. The latter requirement is important to allow distance calculations as described below.

### Normalization of field values

The cell centroids and the bone areas were convoluted with these RBF, which are additive to highlight regions with higher concentrations, respectively, clusters (blue dashed line Fig. 1 for two adjacent nuclei). Since every point has a field with values >0 in every pixel of the image, the summation fields after convolution easily yields high scalar values >10,000 depending on the overall object number. To normalize the scalar fields, the linear mapping

2 $$ {\mathrm{C}}^{\hbox{'}}\left(\mathrm{x}\ \mathrm{y}\right)=\left(\frac{-100}{C_{min}+{C}_{max}}\right)\mathrm{C}\left(\mathrm{x}\ \mathrm{y}\right)+\left(\frac{100}{C_{min}+{C}_{max}}\right){C}_{min} $$

with C(x y) as the local scalar field (equation 1), C_min_ as the absolute minimum and with C_max_ as the absolute maximum of the scalar field is applied.

### Calculation of the gradient and the divergence

One further hypothesis of this work is that the gradient and the divergence of the applied scalar field are meaningful and, respectively, are useful to detect and describe object clusters. This hypothesis is in line with approaches in biophysics, e.g. fluid dynamics [34, 35] and earth science, e.g. digital elevation models [36]. The gradient can be calculated by functions implemented in MATLAB. For example, the normalized gradient is calculated by dividing it by the mean gradient per field according to

3 $$ gradC\left(\mathrm{x}\ \mathrm{y}\right)=\frac{G}{\overline{G}} $$

with C(x y) as the local scalar field (equation 1), G as the gradient of C(x y) and with $\overline{G}$ as the mean gradient of C(x y).The divergence can also easily be calculated by in MATLAB implemented functions and is likewise normalized.

### Statistical evaluation (Additional file 1: Figure S1)

Representation of the field as histograms and statistical evaluation of the distributions were performed in MATLAB. Another assumption is that several readings routinely used for co-localization analysis in fluorescence microscopy could be used for similar analysis with the fields that are produced herein: Calculation of correlation coefficients (Pearson’s correlation coefficient (PCC), overlap coefficient according to Manders (MOC), overlap coefficients M_1_and M_2_ [37, 38]) and graphical presentation were performed with R [39], in particular with the ggplot2 package [40].

## Results

### Proof of principle for the description of colocalization and possible spatial interaction via a scalar field

**Fig. 2:**
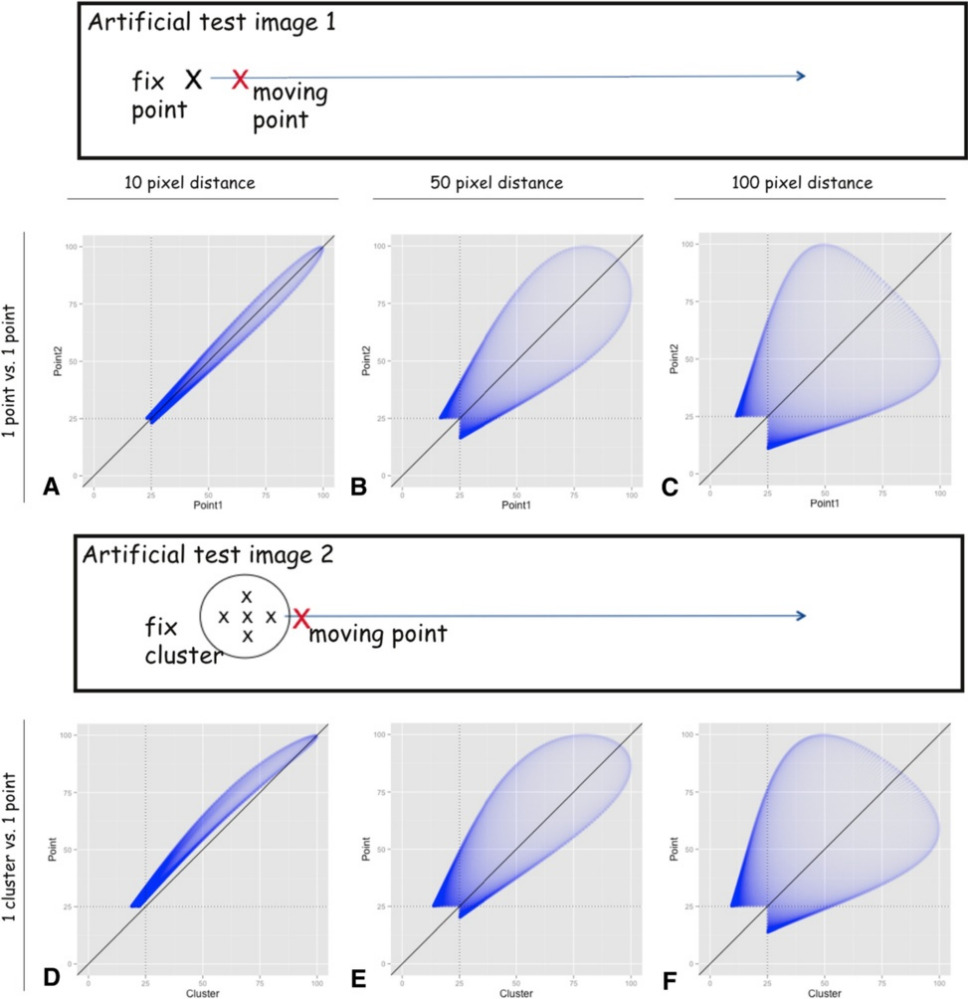
Scatter plots of several artificial test images. Binary test images containing one point and/or a set of points were generated and convoluted with the RBF_direct_. The values of a pair of normalized matrices (compare equation 2) are plotted against each other, resulting in a scatter plot. The sketches above the scatter plots represent the artificial test images (black box) with one fix (black X) and one moving point (red X) and, respectively, one fix cluster of five points (black Xs) and one moving point (red X). For Matlab, these images were realized as matrices of 500x500pixel is one, respectively, five fix points and one moving point (in the supplement). Density values below a threshold of 25 are excluded. **a**-**c**: In the upper line, two matrices each containing one single point are plotted against each other. The position or respectively the distance of these two are changed as described (e.g. point at M(1,1) against N(1,11) etc.). **d-f**: In analogy, one point in the lower line is plotted against one cluster consisting of 5 points

To directly test whether the chosen approach is able to describe proximity of cells or “degree of co-localization” with regard to the above-defined direct interaction, a set of artificial binary test images was analysed. These images were matrices of 500×500pixel that contain one point and/or a set of points; the positions of which were changed in a stepwise manner. The images were convoluted with the above-described RBF_direct_ (hypothesis 1 in the method section that a sharp function could map direct cellular contact) and further processed. The matrices containing the scalar values could be linearized and plotted against each other as routinely performed in co-localization analysis: For a single point versus another single point (Fig. 2a-c) and for a single point versus a cluster (Fig. 2d-f) the width of the point cloud depends on the distance. Furthermore, the shape of the point cloud also depends on the number of points per matrix. Of note, the absolute number of pairs of variants in this case depends on the image size (e.g. for a 10×10 pixel image 100 pairs of variants and not on the number of points.

To characterize these resulting point clouds, several well-established correlation coefficients (e.g. Pearson’s correlation coefficient (PCC) and overlap coefficient according to Manders (MOC) [37, 38]) could be applied after thresholding to avoid false high co-localization due to near zero values (e.g. threshold of 25 in Fig. 2). The PCC and the MOC are inversely correlated with the spatial distance of the points/clusters, since the values of both coefficients decrease with increasing distance: E.g. one point versus one point showed at the distance of 10 pixel PCC = 0.96 and M = 1.00 and accordingly at 100 pixel distance PCC = 0.31 and M = 0.83.

For comparison, without the convolution, the standard colocalization method, at a distance of 0 pixel PCC = 1 and M = 1 and respectively at a distance of 1 pixel PCC = 0 and M = 0.

Furthermore, the established overlap coefficients M_1_ and M_2_ [37, 38] could be obtained after setting a threshold (e.g. 50 to get the overlap of 50-perzentile). These values also correlated with the distance and showed a relation with both the number of points and the distance of points within a cluster: E.g. one cluster and one point at a distance of 50 pixel show Manders coefficients of M_Cluster_ = 0.83 and M_Point_ = 0.59, while the values were M_cluster_ = 0.03 and M_Point_ = 0.02 at a distance of 200 pixel.

### Comparison to colocalization via colour channel analysis

**Fig. 3:**
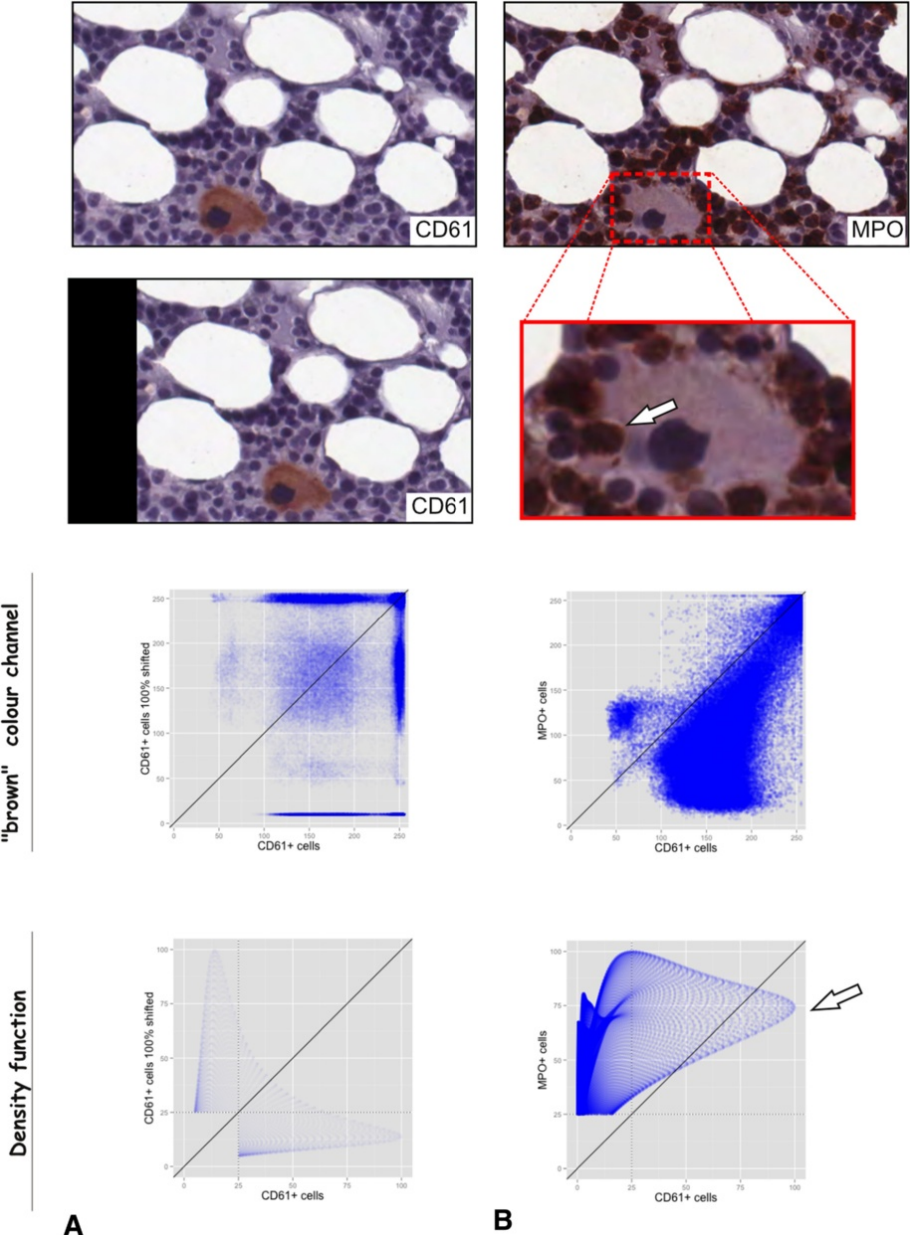
Comparison of the colocalization via RBF to the standard colocalization analysis in fluorescence microscopy. The golden standard of colocalization analysis in fluorescence microscopy is to analyse the different colour channels (e.g. red and green). In this figure we simply matched this method to IHC by comparing and analysing the “brown” channel obtained by colour deconvolution. One small region of a slide sequentially stained for CD61 (A) and MPO (A) was analysed. Subsequently, colocalization on basis of the herein described RBF was compared to analysis of colour chancels. **a** One small region with one CD61-positive megakaryocyte (upper image) was compared to the same region shifted one cell width to left (middle image). The upper scatter plot shows the brown colour channels of the images plotted against each other, whereas the lower one shows the scatter plot for the RBF. **b** The same small region now stained for MPO was used to check for colocalization/direct spatial interaction of the CD61-positive megakaryocyte and the MPO-positive granulocytes (upper image). The arrow in the zoomed part of the image highlight direct spatial interaction of the megakaryocyte and the granulocytes. The upper scatter plot again shows the brown colour channels plotted against each other and the lower one shows the RBFs plotted against each other

The golden standard of colocalization analysis in fluorescence microscopy is to analyse and plot against each other the different colour channels (e.g. red and green) [38]. By comparing and analysing the “brown” channel obtained by colour deconvolution [28], we tried to adapt this approach to IHC images and subsequently compared the results of both methods. First we compared the results for analysis of one CD61-positive megakaryocyte translated one cell width to left (Fig. 3a): Whereas parts of the point cloud are arranged around the bisecting line (Fig. 3a upper scatter plot), the density function-based analysis shows a clearly forked point cloud (lower scatter plot). Second we analysed the same region now stained for MPO by sequential IHC [17] for colocalization of megakaryocytes and granulocytes (Fig. 3b): The point cloud for the colour channel analysis is mostly distributed around the bisecting line (Fig. 3b upper scatter plot). This does not fit to the images where the brown colour is placed in different areas (one brown megakaryocyte in A and many brown granulocytes in B). In comparison, the lower scatter plot shows that there is mostly no correlation between the CD61- and the MPO-positive cells. However, there is one branch of the point cloud that is due to the direct spatial contact of cell populations (Fig. 3b white arrows).

### Proof of principle for valid distance measurements through histogram intersection

The Euclidian distance is usually applied to measure the distance between two objects in an histological sections. Therefore, Euclidian distance is regarded as the gold standard. However, with vast numbers of cells this approach is not feasible for manual counting. Herein, we apply the histogram intersection of scalar fields, which represent objects, as surrogate for distances between different objects. The histogram intersection is widely applied in the comparison of multidimensional distributions [31, 41].

Again, a set of artificial binary test images with variable object distances was analysed: The intersection of the histograms decreased with the distance of two points (for Fig. 2a-c: 0.98, 0.89 and 0.79) and of one point and one cluster respectively (for Fig. 2d-e: 1.00, 0.95 and 0.83). However, due to the shape of the applied RBF (bell-shape with asymptotically approach to zero; please compare equation 1 and Fig. 1), the distance measurement via histogram intersection is only meaningful within a certain range. Beyond a certain distance (for RBF_direct_ approximately 200 pixel/100 μm), the intersection function shows an asymptotic behaviour. Therefore, the distance and the histogram intersection cease correlating beyond a certain value (in this example beyond 0.4).

### Proof of principle for description and detection of object clusters via calculation of the gradient and the divergence

On the basis of the applied scalar fields, several typical values for scalar and vector fields like the gradient (i) and the divergence (ii) could be calculated by standard mathematical operations [42].

Ad (i) The normalized gradient (equation 3) can be used to compare the structure of object clusters in different images regardless of the number of objects.

Ad (ii) In this approach, the object centroids and the clusters are represented by sinks: In an artificial test image containing a cluster of five points with variable distance, it could be observed, that the number of sinks depends on the distance between points: For a distance of 25 pixel or less, the sinks were fused to one single “compound sink” (Additional file 2: Figure S2A), whereas a distance of 125 pixel between each of the 5 points (Additional file 2: Figure S2D) resulted in one sink per point for the present example. These numerical values are examples that are intended to show that the divergence depends on the distance and the shape of the function; and that it can be used to describe clusters of objects.

### Application on histological images

To test the presented approach under less artificial conditions, is was applied to the spatial characterization of lymphoid infiltrates in immunohistochemically stained bone marrow sections using follicular lymphoma as a model for spatially distinct neoplastic lymphoid infiltrates, and chronic myeloid leukaemia as a model of indistinct reactive lymphoid infiltrates among neoplastic cells.

### Example 1: Spatial interaction of B-cell markers in a focal, dense infiltration

Tumour infiltrates of follicular lymphoma in the bone marrow typically form either dense intertrabecular nodules, or dense, linear, paratrabecular lymphoid aggregates along the bone. In sections stained for the markers CD20 and Bcl2, tumorous B cells typical show co-expression [7, 8].

**Fig. 4:**
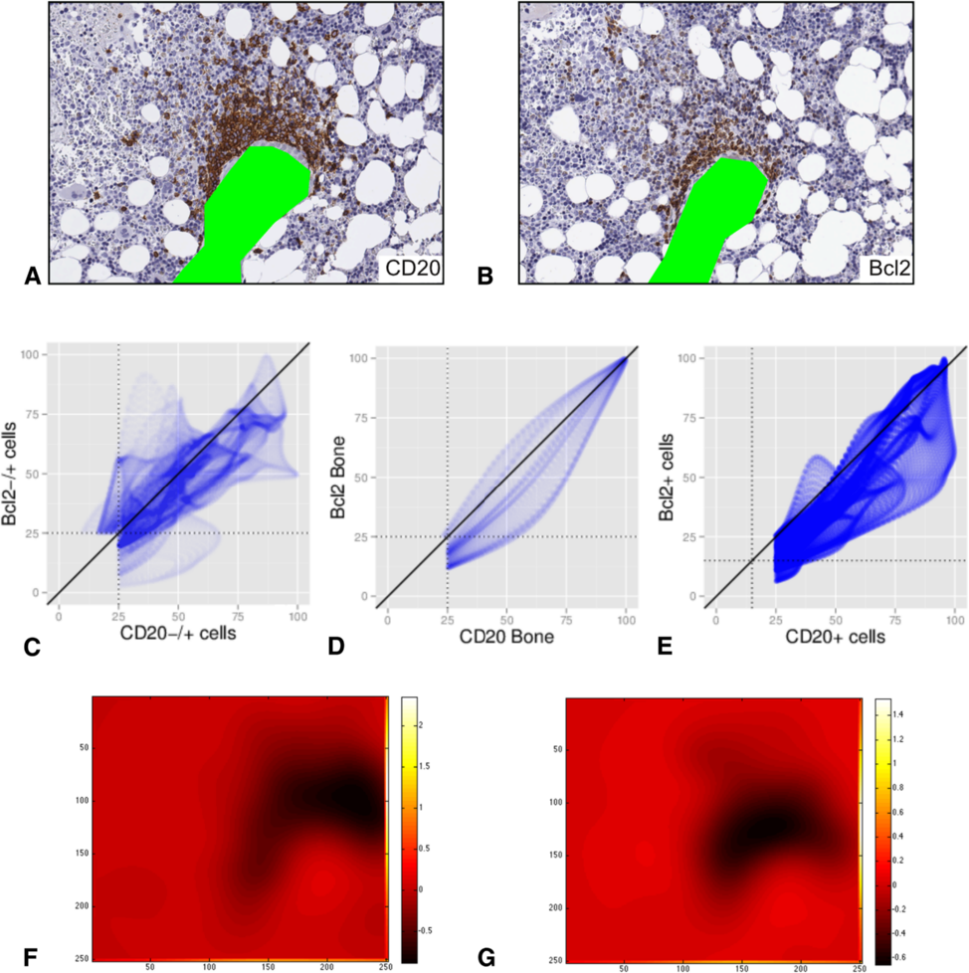
Direct spatial interaction in a case with one focal, dense infiltration. Serial sections of a trephine biopsy from a patient with a follicular lymphoma were stained for CD20 and Bcl2. Slides were fully digitalized, registered and analysed as described in Methods.A-B: Staining for CD20 and Bcl2. After segmentation of nuclei, there were 352 CD20+ and 859 CD20−/+ nuclei in **a**, and 233 Bcl2+ and 795 Bcl2−/+ nuclei in **b.** **c** Scatter plot for all nuclei (CD20−/+ and Bcl2−/+) with PCC = 0.74, MOC = 0.97 and M_CD20−/+_ = 0.90 and M_Bcl2−/+_ = 0.95. **d** Scatter plot for the bone areas with PCC = 0.94, MOC = 0.98 and M_BoneCD20_ = 0.76 and M_BoneBcl2_ = 0.99. **e** Scatter plot for all positive nuclei (CD20+ and Bcl2+) with PCC = 0.90, MOC = 0.97 and M_Bcl2+_ = 0.99 and M_CD20+_ = 0.71, respectively. **f**-**g** Heat map of the diversity for CD20 (F) and Bcl2 (G)

Analysis of the co-localization with RBF_direct_ (Fig. 4f for CD20 and G for Bcl2). Theoretically, the two fields could be combined by addition to one field describing the density of the infiltration by CD20+ Bcl2+ B-cells (data not shown); as delineated in the method section (equation 4).

### Example 2: Allocation and spatial interaction of B- and T-cells in a focal, dense infiltration

Lymphocytosis with lymphoid follicles are a common finding in bone marrow sections [7]. Follicles are usually composed of CD3+ T cells and CD20+ B cells* and are therefore an example for a focal, dense infiltration where immunohistochemical T- and B-cell markers co-localize within the infiltration but not within single cells.

**Fig. 5:**
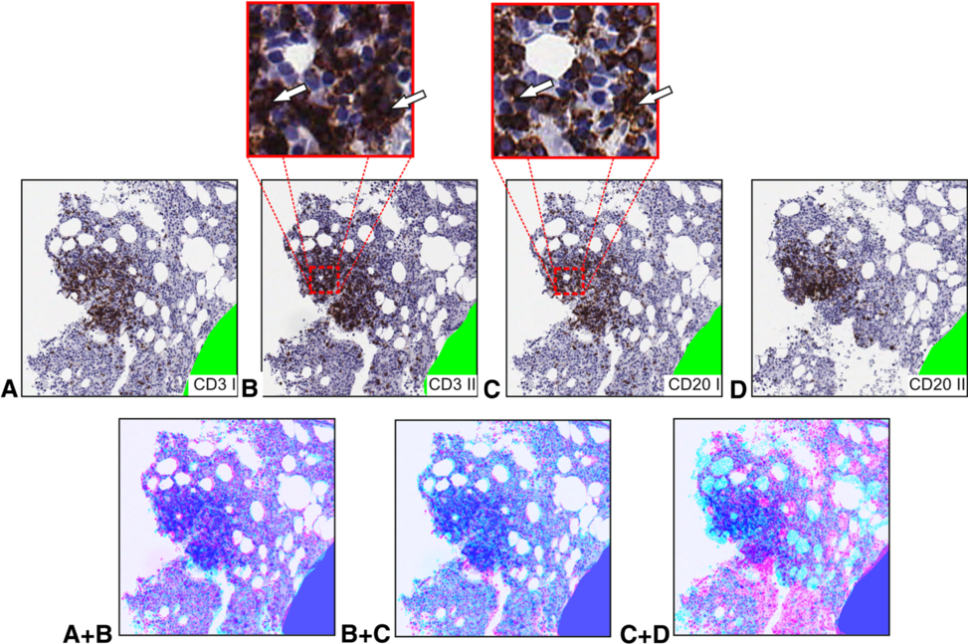
Direct spatial interaction in a lymphoid follicle. Serial sections of a trephine biopsy from a patient with bone marrow lymphocytosis were stained for CD3, CD3, CD20 and CD20 (**a-d**). Subsequently, slides were fully digitalized, registered and analysed as described. By segmentation, there are 320 CD3+ and 1,156 CD3−/+ nuclei in **a**, 392 CD3+ and 1,139 CD3−/+ nuclei in **b**, 316 CD20+ and 1,176 CD20−/+ nuclei in **c** and respectively 202 CD20+ and 1,182 CD20−/+ nuclei in **d**. Overlap readings are calculated for direct interaction (RBF_direct_). A + B: Overlap of both images with A (first section stained for CD3) drawn in red and respectively B (second section stained for CD3) drawn in blue. For stained cells, PCC = 0.95, MOC = 0.99 and M_1st section CD3+_ = 0.98 and M_2nd section CD3_ = 0.81. B + C: Overlap of both images with B (second section stained for CD3) drawn in red and respectively C (first section stained for CD20) drawn in blue. For stained cells, PCC = 0.97, MOC = 0.99 and M_2ndsection CD3+_ = 0.83 and M_1stsection CD20_ = 0.97. C + D: Overlap of both images with C (first section stained for CD20) drawn in red and respectively D (second section stained for CD20) drawn in blue. For stained cells, PCC = 0.67, MOC = 0.92 and M_1st section CD20+_ = 0.57 and M_2nd section CD20_ = 0.97

Analysis of co-localization in serial sections (Fig. 4) stained repeatedly (serially) for CD3 and CD20 (sections CD3 I, CD3 II, CD20 I and CD20 II) showed, that there was a spatial overlap/a co-localization of these markers (Manders coefficients each >0.57). This is against the expectation, since CD3 and CD20 usually are not expressed by one cell. However, a closer look at the images reveals (small insets in Fig. 5), that brown stained areas do co-localize in the images (white arrows in the small insets). Furthermore, from the point of spatial interaction, there is of course an interaction of neighbouring T- and B-cells. Analysing a sketch of a lymphoid follicle composed of B- and T-cells drawn on basis of Fig. 4 carve this point out: In this case there is no overlap of cells but the cells are close neighbours. Therefore M_T-cell_ = 0.34 and M_T-cell_ = 0.67.

### Example 3: Co-localization of T-cell markers in a lose infiltration

In CML, neoplastic myeloid infiltrates in the bone marrow are commonly intermixed with a minority of dispersed lymphocytes of unknown significance [7, 8]. Antibodies to three T cells markers (CD3, CD4 and CD8 [7]) and one B cell marker (CD20) [7] were applied to serial CML bone marrow sections to test for the expected strong overlap between T cell markers (e.g. CD3 and CD8) but for a minor overlap between T and B cell markers (e.g. CD3 and CD20). The resulting overlap coefficients are shown in Table 1 for direct and indirect interaction (furthermore the images and three resulting scatter plots are shown in Additional file 3: Figure S3).

**Table 1:** Comparison of direct and indirect interaction for three registered sections in a case with loose lymphoid infiltrates

	Direct interaction/RBF_direct_								Indirect interaction/RBF_indirect_							
	CD3	CD3 Bone	CD4	CD4 Bone	CD8	CD8 Bone	CD20	CD20 Bone	CD3	CD3 Bone	CD4	CD4 Bone	CD8	CD8 Bone	CD20	CD20 Bone
CD3		M_CD3_ = 0.00	M_CD3_ = 0.00^b^	M_CD3_ = 0.02	M_CD3_ = 0.00^b^	M_CD3_ = 0.02	M_CD3_ = 0.10^a^	M_CD3_ = 0.00		M_CD3_ = 0.60	M_CD3_ = 0.54	M_CD3_ = 0.36	M_CD3_ = 0.54	M_CD3_ = 0.36	M_CD3_ = 0.69^a^	M_CD3_ = 0.60
CD3 Bone	M_CD3Bone_ = 0.00		M_CD3Bone_ = 0.03	M_CD3Bone_ = 0.43^a^	M_CD3Bone_ = 0.00	M_CD3Bone_ = 0.43^b^	M_CD3Bone_=0.00	M_CD3Bone_=0.88	M_CD3Bone_ = 0.89		M_CD3Bone_ = 0.71	M_CD3Bone_ = 0.50	M_CD3Bone_ = 0.71	M_CD3Bone_ = 0.50	M_CD3Bone_=0.59	M_CD3Bone_=0.98
CD4	M_CD4_ = 0.00^b^	M_CD4_ = 0.61		M_CD4_ = 0.00	M_CD4_ = 0.00	M_CD4_ = 0.00	M_CD4_ = 0.00	M_CD4_ = 0.39	M_CD4_ = 0.91	M_CD4_ = 0.80		M_CD4_ = 0.24	M_CD4_ = 0.72	M_CD4_ = 0.21	M_CD4_ = 0.69	M_CD4_ = 0.24
CD4 Bone	M_CD4Bone_ = 0.00	M_CD4Bone_ = 0.79^a^	M_CD4Bone_ = 0.00		M_CD4Bone_ = 0.00	M_CD4Bone_=0.84	M_CD4Bone_=0.01	M_CD4Bone_=0.31	M_CD4Bone_ = 0.88	M_CD4Bone_ = 0.83	M_CD4Bone_ = 0.36		M_CD4Bone_ = 0.66	M_CD4Bone_=0.92	M_CD4Bone_=0.54	M_CD4Bone_=0.82
CD8	M_CD8_ = 0.00^b^	M_CD8_ = 0.00	M_CD8 =_0.00	M_CD8_ = 0.00		M_CD8_ = 0.00	M_CD8_ = 0.00	M_CD8_ = 0.00	M_CD8_ = 0.91	M_CD8_ = 0.80	M_CD8 =_ 0.50	M_CD8_ = 0.31		M_CD8_ = 0.27	M_CD8_ = 0.80	M_CD8_ = 0.51
CD8 Bone	M_CD8Bone_ = 0.00	M_CD8Bone_ = 0.71^b^	M_CD8Bone_ = 0.00	M_CD8Bone_ = 0.96	M_CD8Bone_ = 0.00		M_CD8Bone_=0.01	M_CD8Bone_=0.25	M_CD8Bone_ = 0.89	M_CD8Bone_ = 0.83	M_CD8Bone_ = 0.21	M_CD8Bone_ = 1.00	M_CD8Bone_ = 0.64		M_CD8Bone_=0.51	M_CD8Bone_=0.83
CD20	M_CD20=0.15_^a^	M_CD20=0.00_	M_CD20_ = 0.00	M_CD20_ = 0.06	M_CD20_ = 0.00	M_CD20_ = 0.07		M_CD20_ = 0.00	M_CD20=0.99_^a^	M_CD20=0.57_	M_CD20_ = 0.59	M_CD20_ = 0.31	M_CD20_ = 0.99	M_CD20_ = 0.27		M_CD20_ = 0.58
CD20 Bone	M_CD20Bone=0.00_	M_CD20Bone=0.91_	M_CD20Bone_=0.03	M_CD20Bone_=0.46	M_CD20Bone_=0.00	M_CD20Bone_=0.44	M_CD20Bone_=0.00		M_CD20Bone=0.90_	M_CD20Bone=0.99_	M_CD20Bone_=0.36	M_CD20Bone_=0.50	M_CD20Bone_=0.68	M_CD20Bone_=0.46	M_CD20Bone_=0.60	

Three sequential sections stained for CD3, CD20, CD4 and CD8 were registered and subsequently the overlap coefficients were calculated for direct and indirect interaction (application of RBF_direct_ with its sharp shape and respectively RBF_indirect_ with its broad silhouette)

^a^As expected, small overlap/direct interaction between CD3+ and CD20+ cells and noticeable indirect, paracrine interaction

^b^Unexpected low overlap between the T cell markers CD3, CD4 and CD8 assumedly due to morphological changes in the course of serial sectioning (see arrows in Additional file 3: Figure S3). Modification of the RBF_direct_ would overcome this problem, however, at the cost of reduced specificity

The images are fairly registered with regard to the bone trabeculae (Manders coefficients for bone 0.5-0.92 and for all cells >0.95). The reduced overlap is due to morphological changes that inevitably occur when sectioning through different levels of a core biopsy. As expected and shown in Table 1, there is no direct interaction between CD3+ and CD20+ (M_CD3_ = 0.10 versus M_CD20_ = 0.15). Due to morphological changes (the changes are highlighted by arrows in Additional file 3: Figure S3), there is also no spatial interaction between CD3, CD4 and CD8 (Manders coefficients each 0.00). For the indirect interaction, the Manders coefficients are each higher but still less than for CD3 and CD20. This finding will be picked up in the Discussion.

### Example 4: Localization of cellular infiltrates in relation to bone trabeculae

While the above paragraphs describe the direct spatial interaction/co-localization of certain cell populations or rather stained and non-stained cells, we next addressed the allocation of cells to a pre-defined niche. The periosteal niche is defined as the space in close proximity to bone trabeculae [30, 43, 44]. Therefore, depending on its distance to the closest bone, a given cell or population could be allocated (or not) to this niche.

One way to implicitly address distance is to run a co-localization/interaction analysis on basis of RBF_indirect_. By doing so, for the dense lymphoid infiltration in example 1 (follicular lymphoma) there is more indirect spatial interaction (k_CD20+_ = 0.91) than for the “loose” infiltrate in example 2 (k_CD20+_ = 0.73) between CD20+ cells and the bone trabeculae.

**Fig. 6:**
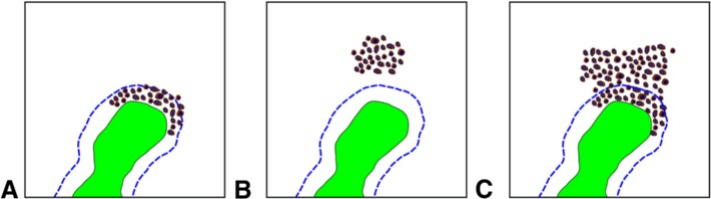
Sketches of different bone marrow infiltration by a lymphoma. Sketch based on the region shown in Fig. 3 to visualize different patterns of bone marrow infiltration. The bone trabeculae are drawn in green. The blue dashed line delineates a region with a maximal sagittal distance of approximately 20 μm around the bone that is regarded as the location of the periosteal niche (compare [50]). Histogram intersection is calculated for direct and indirect interaction (RBF_direct_ and RBF_indirect_). **a** Nodular, peritrabecular infiltration (intersection 0.81 ± 0.05 for direct and 0.88 ± 0.03 for indirect interaction). **b** Nodular, non-peritrabecular infiltration (intersection 0.51 ± 0.05 for direct and 0.77 ± 0.02 for indirect interaction). **c** Diffuse bone marrow infiltration (intersection 0.66 ± 0.013 for direct and 0.82 ± 0.05 for indirect interaction)

Distance could also be measured by another approach, namely by calculating the histogram intersection (as depicted above) for bone trabeculae and a cell population. Drawn on basis of the bone marrow region Fig. 3, the sketches in Fig. 6 show different types of bone marrow infiltration by a lymphoma. For example, 33 cells located only on the blue dashed have a mean histogram intersection of 0.81 ± 0.07 for direct interaction (and of 0.91 ± 0.06 for indirect interaction). Assuming that these values as threshold for allocation into the periostal region, the sketched infiltrations within Fig. 4 could be analysed accordingly: The mean histogram intersection for the dense infiltration in A is 0.81 ± 0.05 (respectively 0.88 ± 0.03 for indirect interaction), while it is 0.51 ± 0.05 (respectively 0.77 ± 0.02) for the non-paratrabecular infiltration in B, and 0.66 ± 0.013 (respectively 0.82 ± 0.05) for the diffuse, mixed infiltration in C. Hence, the infiltration in Fig. 4a could be allocated by the histogram intersection to the periostal region. Applying the intersection values of cells on the blue line as threshold (0.81 ± 0.07), object per object revealed a significantly different mean intersection of 0.82 ± 0.07 for the dense infiltration in example 1 compared to 0.77 ± 0.15 for the “loose” infiltrate in example 2 (*p* = 0.017).

## Discussion

The herein presented approach (compare Additional file 1: Figure S1) enables to run morphological analysis of bone marrow infiltrates, which is a demanding task due to – amongst other – highly complex immunophenotypes (e.g. Treg are defined by CD4 CD25 and FoxP3). Immunohistochemical staining of serial sections or sequential IHC can be performed to detect such cells that are defined by a marker set. (1) Herein we present an approach to propagate the cell position through serial slides by a scalar field function (Fig. 1a task A1). By doing so colocalization methods established for immunofluorescence could by applied. (2) Besides this, the scalar field function introduces a new perspective to objectively and automatically interpret complex objects (e.g. cellular infiltrates) and different modes of their spatial relationships (e.g. direct vs. paracrine) in histological sections (Fig. 1 task A2). Furthermore it allows for (3) a qualitative assessment of e.g. direct cellular contact or paracrine contact (Fig. 1 task A3) and by doing so also enables to (4) detect cell clusters.

### 1) Is co-localization analysis on basis of scalar fields in serial sections possible?

The co-localization/spatial interactions (direct or indirect) could be measured by using several well-established correlation coefficients (e.g. Manders’ overlap coefficient [37]) and by overlap coefficients M_1_ and M_2_ [38]. However, since these methods are initially defined for scatter plots of intensity values of different channels (e.g. green and red) and not for scalar field values, the interpretation of the resulting graphs and values need to be adapted: There is no relation between the number of particles and the points in the scatter plot similar to a scatterplot of two colour channels of one immunofluorescence image. The shape and position of the point cloud encodes the co-localization and also the clustering (see Fig. 2). In this context, especially the overlap coefficients M_1_ and M_2_ seem to fit best for the spatial interaction/co-localization analysis; by setting a threshold, one obtains the overlap of the corresponding percentiles (e.g. 50 %-percentile in Fig. 3 and Additional file 3: Figure S3).

At first glance, one may assume that it is a drawback of the approach described here that absolute distance values of single cells and clusters (e.g. in [m]) are lost. However, it is possible to state the overlap in form of overlap coefficients (e.g. Manders overlap coefficients M_1_ and M_2_) or as histogram intersection. These measurements may be more significant in regard to biology as pure metric measurements, since they already incorporate interaction models depending on distances for direct and indirect cellular interaction [33].

### 2) Is there are difference between co-localization and spatial interaction?

Throughout this work, the terms co-localization and spatial interaction are used more or less synonymously: According to their shape the scalar fields could describe different interactions models with (hypothesis 1) RBF_direct_ for spatial object-object interaction (maximum value at the centroid, medium values at the cell edge and then a step slope; compare black solid line in Fig. 1b) and (hypothesis 2) RBF_indirect_ for indirect interaction (medium values within the proposed 250 μm range for paracrine interaction [33, 34] and a slight slope; compare red solid line in Fig. 1b); these fields could overlap and, therefore, could describe co-localization on basis of overlap. Thus, an huge overlap of RBF_direct_ points to spatial co-localization of two objects whereas an intermediate (e.g. histogram intersection of >0.95 for direct spatial contact and of 0.81 for 20 μm distance) overlap rather points to a close neighbourhood of them. Whether two objects are co-localized or occur as neighbours mainly depends on the chosen shape of the radial basis function; a broad radial basis function is less prone to registration errors whereas a narrow function is more specific for real overlap of objects. This trade off comes to effect in example 3 and in the linked sketch in Additional file 4: Figure S4: On the one hand, the narrow RBF results in very specific co-localization of markers; on the other hand this function is very prone to morphological changes throughout serial sections. This limitation can almost certainly be overcome by applying repetitive cycles of staining/de-staining using a spectrum of different antibodies on one given section [17, 45–47].

### 3) What are the advantages of the presented field approach?

After segmentation of e.g. cell nuclei in an image by standard image processing techniques, there are usually some distributed or clustered objects in the resulting image. To describe this distribution, clustering analysis could be applied [48, 49]. Therefore, a distance (usually spatial distance) measurement needs to be formulated and subsequently objects within a certain distance are (or not) assigned to a cluster. Although this approach appears straightforward and comprehensive, it depends on each single cell and is therefore prone to image artefacts and registration errors.

To avoid this disadvantage of spatial distance measurement, we herein describe each object (e.g. cell nucleus) by a scalar field on the basis of an inverse multiquadric radial basis function. These fields can overlap and interact in such a way, that the scalar values are summed. Consequently, not single objects but populations represented by a summation field are subject to the subsequent analysis, making this approach more robust against e.g. registration errors. The resulting field can be propagated through several matched sections of one tissue and can therefore be used to analyse co-localization and spatial interaction, notwithstanding the fact that a given single cell may not be represented at the same location in all slides due to image registration errors or due to section thickness. Furthermore, not only the position of a cell could be propagated and described: In the future we plan to describe more abstract properties of objects, such as relative object sizes, as field, that can be propagated and analysed subsequently, respectively. Furthermore, the description as scalar field will allow to use the whole body of field theory in future analyses.

### 4) Is it possible to describe clustering via scalar fields?

A great advantage of scalar fields is that they code the number of objects and their clustering. By calculating on this basis the gradient field and especially its divergence, cell clusters can be described as sources/sinks (compare Additional file 2: Figure S2). This approach does not need an explicit definition of a cluster e.g. by measuring absolute distances, but does implicitly define clusters by the shape of the RBF. Again, in contrast to direct measurements, this may incorporate already quasi-functional aspects by applying RBFs with different silhouettes according to different modes of interaction.

## Conclusions

The description of objects in a histological section as scalar fields (e.g. cells of a certain type) opens several new perspectives: i) Co-localization of different marker (e.g. immunohistochemical staining) in serial sections becomes feasible. ii) Different types of spatial interaction (e.g. direct cellular and paracrine communication) could be modelled and subsequently analysed. iii) Description of aggregated objects via summation fields leads to an implicitly cluster definition.

## Additional files

Additional file 1: Figure S1. Flow chart for the presented approach. The main steps are “preprocessing” in Fiji, “cell segmentation” in Matlab, “introduction of the RBF” in Matlab and “statistical evaluation” in Matlab and R. The preprocessing and the cell segmentation are performed by custom arranged standard methods like colour deconvolution, thresholding, cross correlation etc. (PNG 220 kb) Additional file 2: Figure S2. Heat map of diversity for a cluster of five points. A cluster of five points is convoluted with the RBF_direct_ to obtain a scalar field. For this field the gradient field and - after normalization (compare equation 3) hereof - the diversity were calculated. In this approach, a sink represents a centroid. The distance of the points composing the cluster is changed from 25 pixel in A, to 38 pixel in B, to 50 pixel in C and to 125 pixel in D. (PNG 177 kb) Additional file 3: Figure S3. Direct spatial interaction in a case with a loose infiltration. Sections from a patient with CML were stained for CD3, CD20, CD4 and CD28. Slides were fully digitalized and registered like in the method section described. After segmentation there were 417 CD3+ and 6,187 CD3−/+ nuclei; 17 CD20+ and 5,770 CD20−/+ nuclei; 57 CD4+ and 6,394 CD4−/+ nuclei; and respectively 126 CD8+ and 5,696 CD8−/+ nuclei. A: Registered sections IHC-stained for CD3, CD20, CD4 and CD8. The registration between CD3 and CD20 and respectively between CD4 and CD8 is visually pretty good. However, due to different cuttings levels during processing, there is a continuous change of morphology. The white and the yellow arrow visualize these changes for one trabeculae and, respectively, for a focal lymphoid infiltrate. B: Scatter plot for all nuclei (CD3−/+, CD20−/+, CD4−/+ and CD8−/+). For CD3 vs. CD20 M_CD3−/+_ = 0.90 and M_CD20−/+_ = 0.90; for CD3 vs. CD4 M_CD3−/+_ = 0.90 and M_CD4−/+_ = 0.79; and respectively for CD4 vs. CD8 M_CD4−/+_ = 0.95 and M_CD8−/+_ = 0.80. C: Scatter plot for positive nuclei (CD3+, CD20+, CD4+ and CD8+). The respective overlap coefficients are shown in Table 1. (PNG 3007 kb) Additional file 4: Figure S4. Sketch of a lymphoid follicle. Sketch based on the region shown in Fig. 4 to visualize a non-malignant lymphoid follicle, which is composed of a mixture of T and B cells. A: T cells. B: B cells. (PNG 41 kb)

## Abbreviations

CD: Cluster of differentiation
CML: Chronic myeloid leukemia
Foxp3: Forkhead box P3
MOC: Mander’s overlap coefficient
MPO: Myeloperoxidase
PCC: Pearson’s correlation coefficient
RBF: Radial basis function
Treg: Regulatory T-cell
