# Supplementary figures and images for: On the representation of cells in bone marrow pathology by a scalar field: propagation through serial sections, co-localization and spatial interaction analysis
Source: Diagn Pathol. 2015 Sep 2;10:151. doi: 10.1186/s13000-015-0383-0 (PMC4557224; doi:10.1186/s13000-015-0383-0)

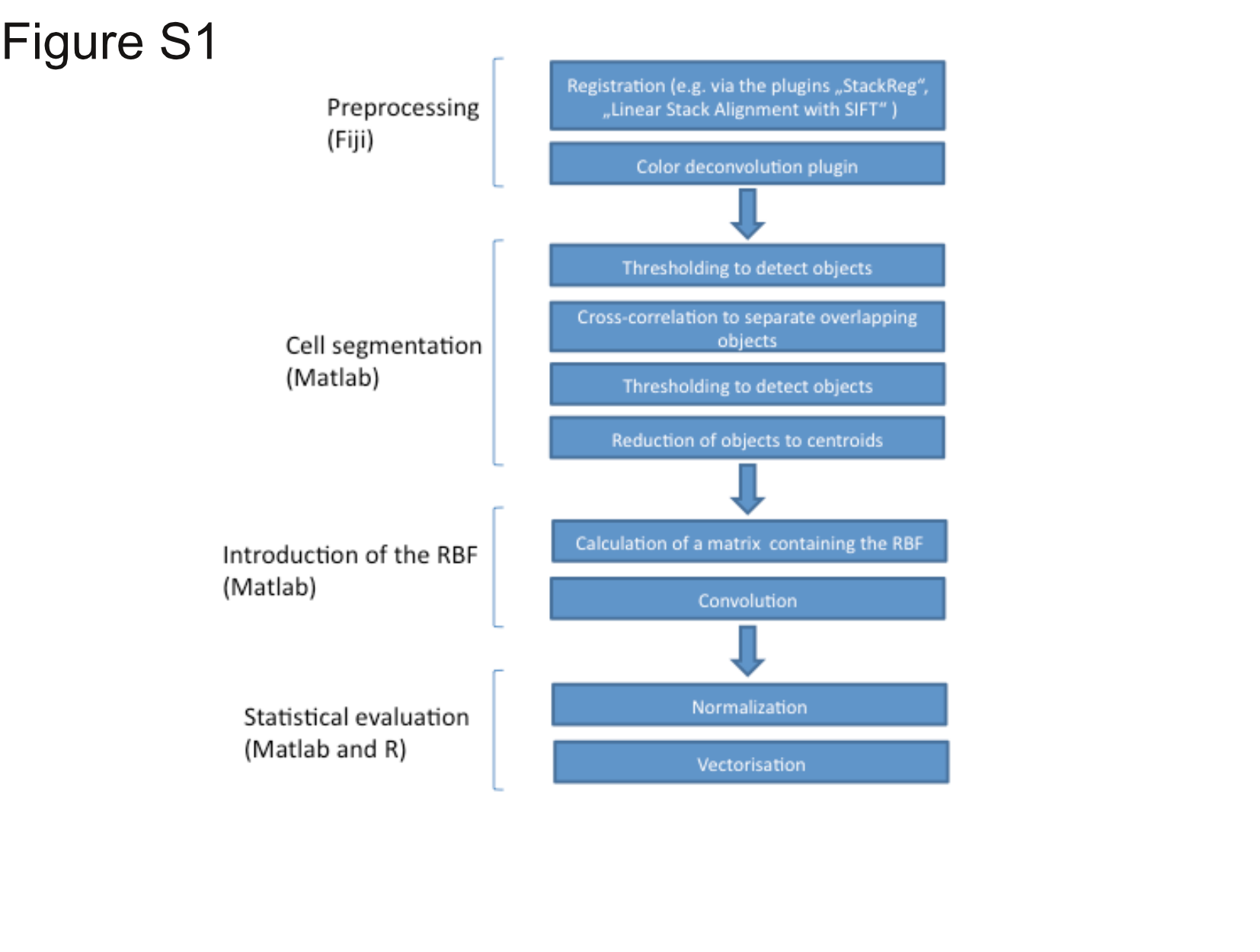

Supplement: Additional file 1: Figure S1. — Flow chart for the presented approach. The main steps are “preprocessing” in Fiji, “cell segmentation” in Matlab, “introduction of the RBF” in Matlab and “statistical evaluation” in Matlab and R. The preprocessing and the cell segmentation are performed by custom arranged standard methods like colour deconvolution, thresholding, cross correlation etc. (PNG 220 kb) [file 13000_2015_383_MOESM1_ESM.png]

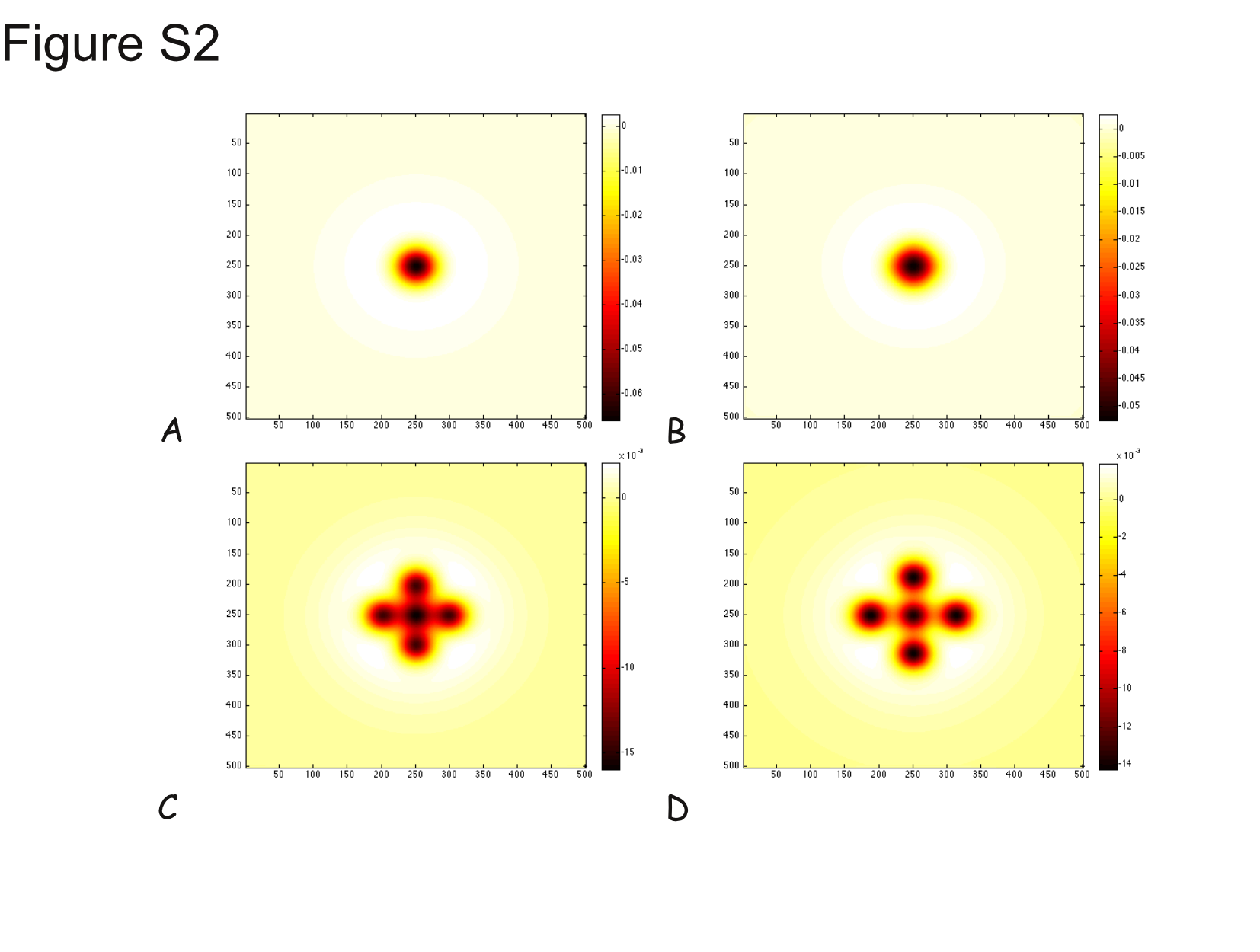

Supplement: Additional file 2: Figure S2. — Heat map of diversity for a cluster of five points. A cluster of five points is convoluted with the RBFdirect to obtain a scalar field. For this field the gradient field and - after normalization (compare equation 3) hereof - the diversity were calculated. In this approach, a sink represents a centroid. The distance of the points composing the cluster is changed from 25 pixel in A, to 38 pixel in B, to 50 pixel in C and to 125 pixel in D. (PNG 177 kb) [file 13000_2015_383_MOESM2_ESM.png]

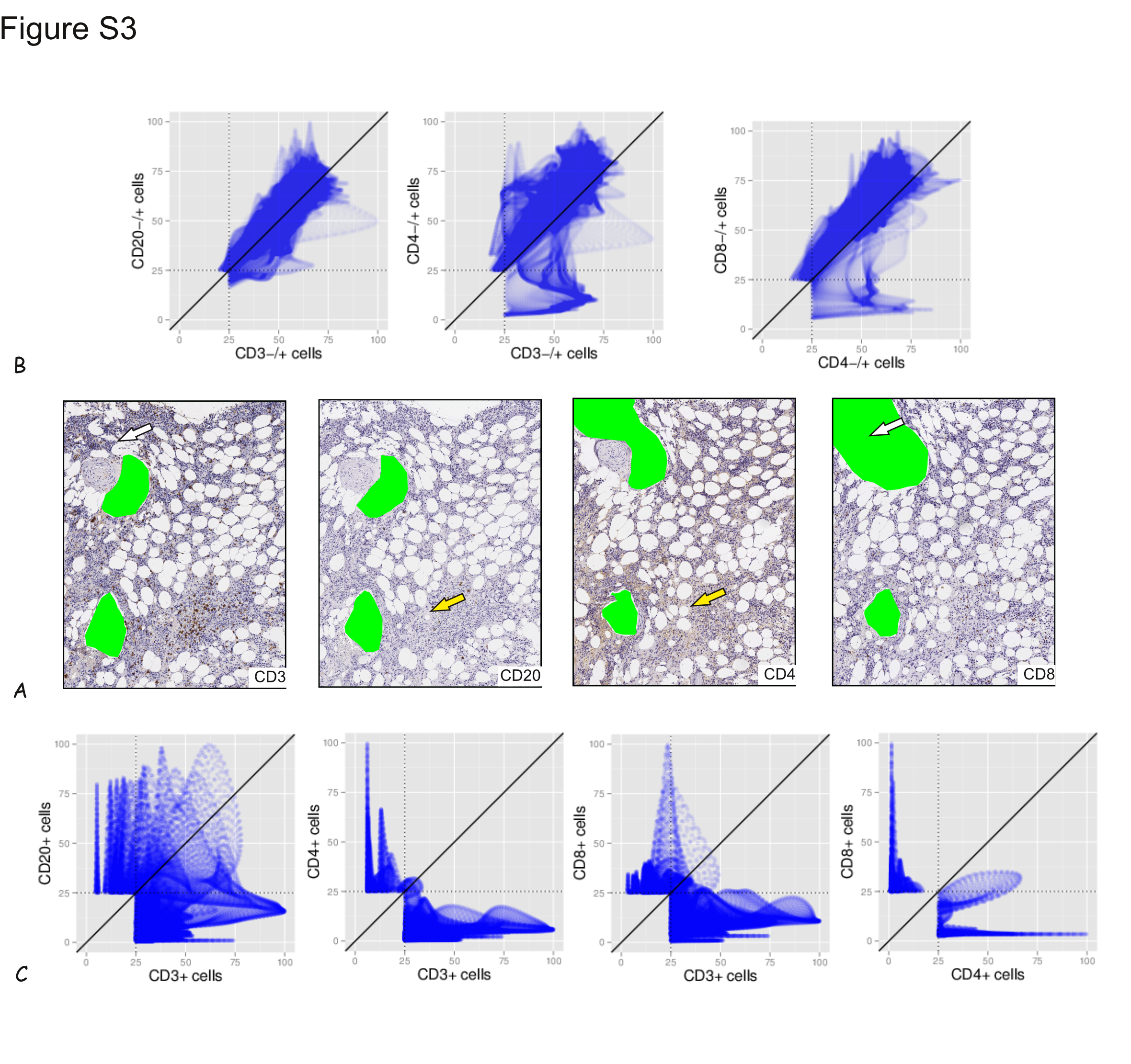

Supplement: Additional file 3: Figure S3. — Direct spatial interaction in a case with a loose infiltration. Sections from a patient with CML were stained for CD3, CD20, CD4 and CD28. Slides were fully digitalized and registered like in the method section described. After segmentation there were 417 CD3+ and 6,187 CD3−/+ nuclei; 17 CD20+ and 5,770 CD20−/+ nuclei; 57 CD4+ and 6,394 CD4−/+ nuclei; and respectively 126 CD8+ and 5,696 CD8−/+ nuclei. A: Registered sections IHC-stained for CD3, CD20, CD4 and CD8. The registration between CD3 and CD20 and respectively between CD4 and CD8 is visually pretty good. However, due to different cuttings levels during processing, there is a continuous change of morphology. The white and the yellow arrow visualize these changes for one trabeculae and, respectively, for a focal lymphoid infiltrate. B: Scatter plot for all nuclei (CD3−/+, CD20−/+, CD4−/+ and CD8−/+). For CD3 vs. CD20 MCD3−/+ = 0.90 and MCD20−/+ = 0.90; for CD3 vs. CD4 MCD3−/+ = 0.90 and MCD4−/+ = 0.79; and respectively for CD4 vs. CD8 MCD4−/+ = 0.95 and MCD8−/+ = 0.80. C: Scatter plot for positive nuclei (CD3+, CD20+, CD4+ and CD8+). The respective overlap coefficients are shown in Table 1. (PNG 3007 kb) [file 13000_2015_383_MOESM3_ESM.png]

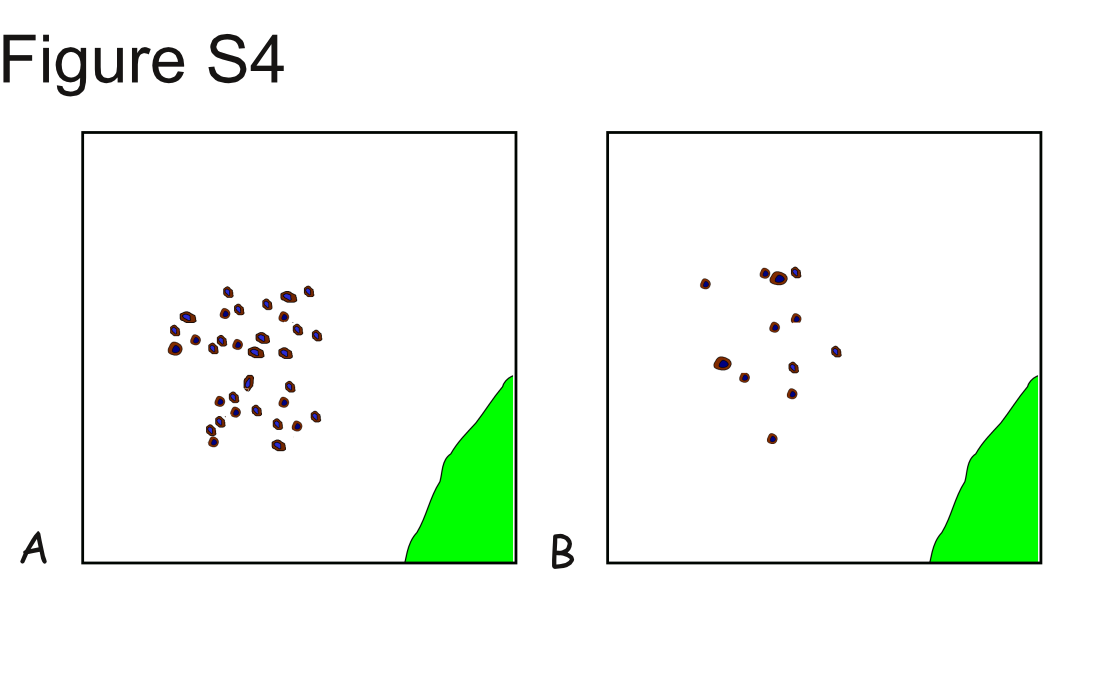

Supplement: Additional file 4: Figure S4. — Sketch of a lymphoid follicle. Sketch based on the region shown in Fig. 4 to visualize a non-malignant lymphoid follicle, which is composed of a mixture of T and B cells. A: T cells. B: B cells. (PNG 41 kb) [file 13000_2015_383_MOESM4_ESM.png]
